# Obstructive sleep apnoea and glucose dysregulation in type 2 diabetes: a systematic review of the literature

**DOI:** 10.3389/fmed.2026.1915032

**Published:** 2026-09-07

**Authors:** Karlygash Shinalieva, Marina Ivanova, Serik Irimbetov, Telman Seisembekov

**Affiliations:** 1 Department of Neurology, NCJSC “Astana Medical University”, Astana, Kazakhstan; 2 Department of Anesthesiology, Intensive Care and Emergency, NCJSC “Astana Medical University”, Astana, Kazakhstan; 3 Heart Rhythm Research Institute, NCJSC “Astana Medical University”, Astana, Kazakhstan

**Keywords:** glucose dysregulation, insulin resistance, intermittent hypoxia, metabolic dysfunction, obstructive sleep apnoea, type 2 diabetes mellitus

## Abstract

**Background:**

Obstructive sleep apnoea (OSA) and type 2 diabetes mellitus (T2DM) are common chronic conditions with a potentially bidirectional relationship mediated by insulin resistance, intermittent hypoxia, and systemic inflammation. However, evidence linking OSA with glucose dysregulation remains inconsistent.

**Objective:**

To evaluate the association between OSA and glucose dysregulation, including prediabetes and T2DM, and related metabolic and cardiovascular outcomes. Methods: A PRISMA-based systematic review was conducted using major databases.

**Results:**

In total, eleven studies from Asia, Europe, Africa, and South America were included in this review. The results show that OSA was consistently associated with poorer glycemic control, increased insulin resistance, and adverse metabolic profiles, with several studies demonstrating severity-dependent effects. In addition, poor sleep quality and short sleep duration were also linked to adverse metabolic outcomes, although findings were less consistent. Finally, limited evidence suggested that continuous positive airway pressure (CPAP) therapy may improve some metabolic and renal outcomes.

**Conclusion:**

OSA is consistently associated with impaired glucose metabolism and increased cardiometabolic risk in individuals with T2DM. Further longitudinal and interventional studies are needed to clarify causal pathways and the metabolic effects of OSA treatment.

**Systematic review registration:**

PROSPERO (CRD420261402284).

## 1 Introduction

Obstructive sleep apnoea (OSA) and type 2 diabetes mellitus (T2DM) are prevalent chronic disorders that represent major clinical and public health burdens worldwide (1). Approximately 830 million adults worldwide are living with diabetes, with more than 90% of cases attributable to T2DM, and the prevalence continues to increase, particularly in low- and middle-income countries (1, 2). Similarly, OSA is now recognized as one of the most common sleep disorders, affecting an estimated 936 million adults aged 30–69 years globally, with approximately 425 million individuals experiencing moderate-to-severe disease requiring clinical evaluation or treatment (3). Both disorders are associated with considerable morbidity, increased healthcare utilization, reduced quality of life, and premature mortality, underscoring their importance as major global public health challenges (2–4).

A number of epidemiological studies have demonstrated a strong bidirectional association between these conditions, with OSA being frequently observed among individuals with T2DM, and T2DM also occurring more commonly in patients with OSA (2). Although both disorders share established risk factors, particularly obesity and advanced age, emerging evidence suggests that their relationship is not solely explained by shared susceptibility. Instead, there is increasing recognition that OSA and T2DM may contribute to each other’s onset and progression. For example, in OSA, recurrent episodes of partial or complete upper airway obstruction during sleep result in intermittent hypoxia and sleep fragmentation, which activate pathophysiological pathways such as sympathetic activation, systemic inflammation, oxidative stress, and HPA axis dysregulation of neuroendocrine systems such as the hypothalamic–pituitary–adrenal (HPA) axis (3, 4).

These mechanisms are thought to disrupt glucose homeostasis by promoting insulin resistance and impairing glucose tolerance, thereby increasing susceptibility to T2DM. Evidence from large cohort studies suggest that OSA is associated with an increased risk of T2DM incident, with a magnitude of effect comparable to traditional metabolic risk factors such as obesity, family history, and physical inactivity. Conversely, T2DM may also contribute to the development and progression of OSA through several mechanisms, including diabetic peripheral neuropathy affecting upper airway neuromuscular control, microvascular dysfunction, increased oxidative and nitrosative stress, as well as impaired central respiratory regulation (5, 6). Despite these proposed mechanisms, the exact biological pathways linking the two conditions remain incompletely defined.

Given this complex bidirectional relationship, there is increasing clinical interest in whether effective management of one condition may influence the course of the other. In particular, clarifying the metabolic effects of OSA treatment in individuals with T2DM, as well as the impact of glycemic status on sleep-disordered breathing, may provide important insights into shared pathophysiology and support integrated therapeutic approaches.

Despite growing research in this area, evidence regarding the association between OSA and prediabetes remains inconsistent, with variability in reported effect sizes and study methodologies. Furthermore, a comprehensive synthesis of the available evidence across the spectrum of glucose dysregulation has not yet been undertaken. Therefore, the aim of this systematic review was to analyze the available evidence on the association between OSA and glucose dysregulation, including prediabetes and T2DM. We further aimed to compare the prevalence and risk of abnormal glucose metabolism between individuals with and without OSA. We hypothesized that OSA is associated with a significantly higher prevalence and risk of both prediabetes and T2DM, independent of shared risk factors such as obesity and age.

## 2 Methods

### 2.1 Data sources and search strategy

This systematic review was conducted in accordance with PRISMA guidelines, and prospectively registered on PROSPERO with the registration number CRD420261402284. The search strategy was developed to identify studies examining sleep disorders (particularly OSA) and glucose dysregulation, including prediabetes and T2DM. Sleep-related terms were identified using a combination of keywords and Medical Subject Headings (MeSH), including sleep disorders, sleep disturbance, insomnia, obstructive sleep apnea, sleep apnea, restless legs syndrome, circadian rhythm disorders, excessive daytime sleepiness, and sleep quality. Diabetes-related terms included T2DM, T2DM, non-insulin-dependent diabetes mellitus, glycemic control, and HbA1c. The search strategy was informed by indexing patterns from previously published studies and relevant systematic reviews.

A comprehensive search was performed in MEDLINE, Embase, Scopus, Web of Science, CINAHL, Cochrane Central Register of Controlled Trials (CENTRAL), PsycINFO, AMED, the Cochrane Database of Systematic Reviews, and the Database of Abstracts of Reviews of Effects (DARE), from database inception to 19 April 2026. In addition, reference lists of all included studies and relevant review articles were manually screened to identify additional eligible publications. The detailed search strategy for each database is provided in Supplementary Table S1.

### 2.2 Study selection and eligibility criteria

All records identified through database searching were screened initially by one of three reviewers based on title relevance. Potentially eligible studies were subsequently screened independently by two reviewers based on abstracts using predefined inclusion and exclusion criteria. Full-text articles were retrieved for studies considered potentially eligible by either reviewer. The same two reviewers independently assessed the full-text articles for final inclusion, with any disagreements resolved through discussion or consultation with a third reviewer.

Studies were included if they met all of the following criteria: (i) involved adults aged ≥18 years with T2DM, prediabetes, or a comparison population without diabetes; (ii) evaluated one or more sleep disorders or sleep disturbances, including OSA, insomnia, restless legs syndrome, circadian rhythm disorders, excessive daytime sleepiness, poor sleep quality, or sleep-disordered breathing; (iii) reported at least one quantitative outcome relevant to the review objectives, including glycemic control (e.g., HbA1c), prevalence or incidence of sleep disorders, metabolic or cardiovascular outcomes, diabetes-related complications, mental health outcomes, quality of life, hospitalization, or mortality; and (iv) were published within the last decade to reflect contemporary clinical practice and current evidence. Eligible study designs included observational studies (cross-sectional, cohort, and case–control studies) and randomized controlled trials conducted in hospital, outpatient, primary care, community, or home-based settings.

Studies were excluded if they involved pediatric populations (<18 years), included participants with type 1 diabetes mellitus or gestational diabetes without separately reporting T2DM data, did not assess a sleep disorder or sleep-related exposure, failed to report relevant quantitative outcomes, were review articles, editorials, conference abstracts, case reports, or non-English publications, or provided insufficient data for extraction and analysis.

### 2.3 Quality assessment

The methodological quality of the included studies was independently assessed by two reviewers using standardized risk-of-bias tools according to study design. Randomized controlled trials were evaluated using the Cochrane Risk of Bias 2 (RoB-2) tool, which assesses bias arising from the randomization process, deviations from intended interventions, missing outcome data, outcome measurement, and selection of the reported results. Observational studies (cross-sectional and cohort studies) were assessed using the Newcastle-Ottawa Scale (NOS), adapted for cross-sectional studies where appropriate. The NOS evaluates study quality based on participant selection, comparability of study groups, and outcome assessment. Any disagreements between reviewers were resolved through discussion or consultation with a third reviewer. Studies scoring 7–9 points on the NOS were considered high quality, 5–6 moderate quality, and <5 low quality.

### 2.4 Sensitivity analyses

A structured sensitivity assessment was conducted to evaluate the robustness of the narrative synthesis findings across key outcomes, including glycemic control, cardiovascular outcomes, and diabetes-related complications. Given that a quantitative meta-analysis was not performed due to substantial heterogeneity in study designs, populations, and outcome definitions, a structured robustness assessment was conducted to evaluate consistency of findings across key methodological domains. For example, the influence of study design was examined by comparing findings from cross-sectional studies, cohort studies, and randomized controlled trials to assess the stability of observed associations across different levels of evidence. Also, the impact of study quality was evaluated by considering whether findings were consistent when lower-quality studies or those with limited adjustment for confounders were compared with higher-quality studies. Similarly, the robustness of findings was assessed by examining consistency between studies using objective sleep assessment methods (e.g., polysomnography or laboratory-based measures) and those relying on self-reported or questionnaire-based assessments. The influence of population characteristics was considered by comparing findings from studies conducted in clinical versus population-based settings and across different geographic regions. Finally, consistency of results was evaluated after excluding studies in which sleep disorders or T2DM were not the primary focus, to ensure that mixed-population or secondary analyses did not disproportionately influence the overall interpretation.

## 3 Results

**Figure 1:**
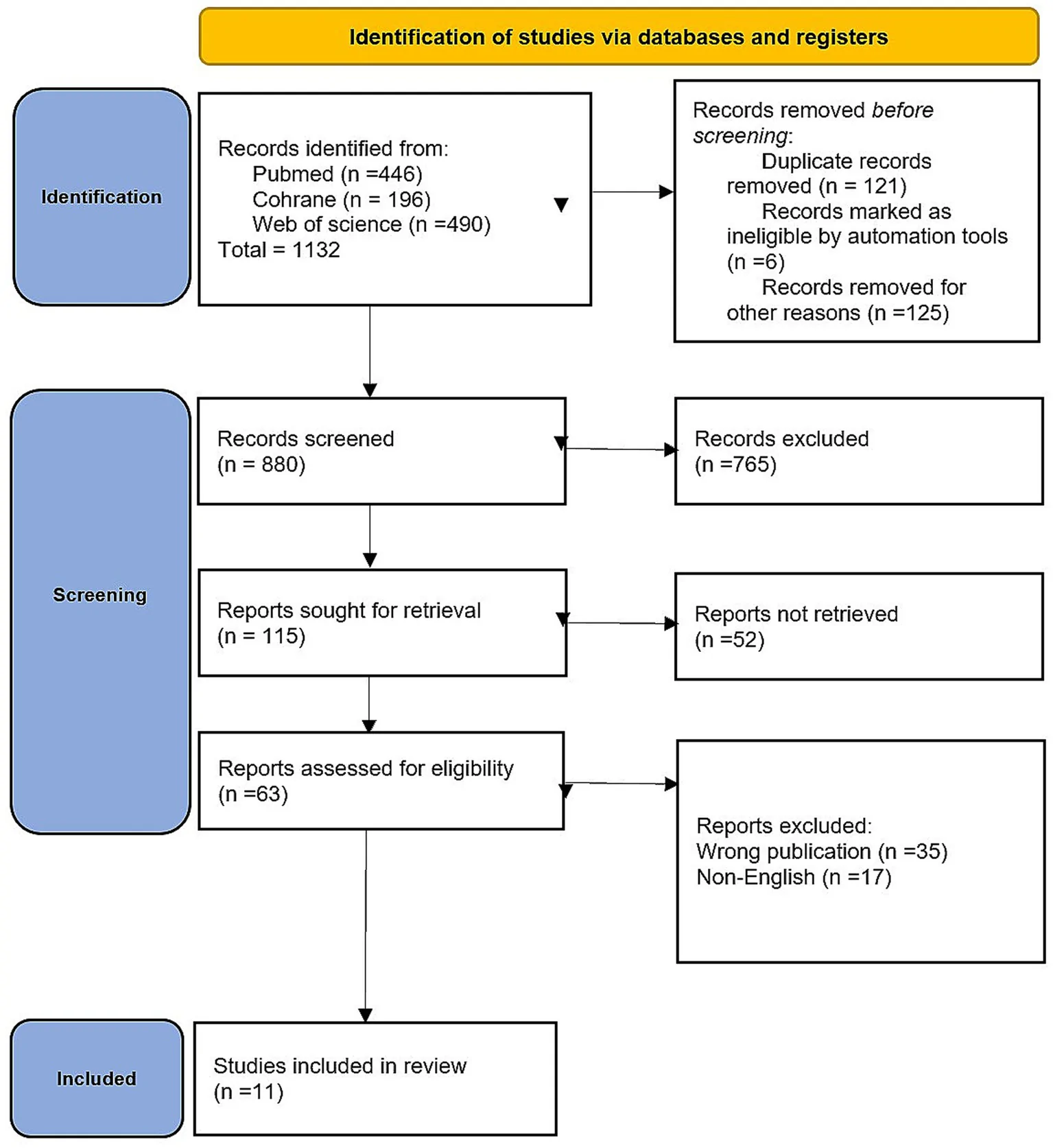
PRISMA flow diagram. The literature search identified 1,132 records from PubMed, Cochrane Library, and Web of Science. After removal of duplicates and other ineligible records, 880 records were screened, and 115 full-text reports were sought for retrieval. Following eligibility assessment, 11 studies met the inclusion criteria and were included in the systematic review. The main reasons for exclusion were inappropriate publication type and non-English language.

The search initially identified 1,132 records through database searching, of which 467 were considered potentially relevant and retrieved for further evaluation. After full-text screening, studies that met the predefined eligibility criteria were included in the review (Figure 1). Reasons for exclusion included ineligible population, absence of sleep disorder assessment, missing outcome data, or unsuitable study design.

Overall, 11 studies met the inclusion criteria and were included in the systematic review. The evidence base comprised eight cross-sectional studies (72.7%), one population-based cohort study (9.1%), and two randomized controlled trials (18.2%). The included studies represented a diverse and methodologically heterogeneous evidence base examining sleep disorders in adults with T2DM. The majority of studies were observational in design, with cross-sectional studies forming the largest proportion (7–14), followed by one population-based cohort study (15) and two randomized controlled trials (16, 17) (Table 1).

**Table 1:** Characteristics of studies included in systematic review.

Author	Year	Country	Study design	OSA diagnostic tool	Sample size	OSA prevalence (%)	Average age	Men (%)	Mean BMI	Main findings
Kalakattawi et al. (7)	2017	Saudi Arabia	Cross-sectional	Berlin Questionnaire, STOP-Bang	270	44–52% high risk	56 years	45%	33 kg/m^2^	High OSA risk associated with obesity and poor glycemic control
Sokwalla et al. (8)	2017	Kenya	Cross-sectional comparative	Berlin Questionnaire, PSQI	223	44% high risk	59 years	48%	27 kg/m^2^	Poor sleep quality and elevated OSA risk in T2DM
Viswanathan et al. (9)	2017	India	Cross-sectional	Berlin Questionnaire/PSG referral	234	60%	53 years	61%	29 kg/m^2^	Very high prevalence of OSA among diabetic patients
Westlake et al. (10)	2016	Czech Republic	Prospective	Berlin Questionnaire, STOP-Bang, PSG	65	49%	60 years	66%	32 kg/m^2^	STOP-Bang showed higher sensitivity for OSA screening
Zhang et al. (11)	2016	China	Cross-sectional	Overnight polysomnography	880	60–67%	57 years	63%	28 kg/m^2^	OSA strongly associated with metabolic dysfunction
Liu & Wu (15)	2017	Taiwan	Population cohort	Clinical diagnosis/database coding	>30,000	Not reported	Adults	Not reported	Not reported	Demonstrated bidirectional association between OSA and diabetes
Li et al. (12)	2020	China	Cross-sectional	Polysomnography	422	56%	30 years	70%	31 kg/m^2^	OSA worsened glucose dysmetabolism and β-cell dysfunction
Michalek-Zrabkowska et al. (13)	2021	Poland	Cross-sectional	Polysomnography	102	100% (OSA cohort)	53 years	73%	31 kg/m^2^	OSA independently associated with insulin resistance
Silva et al. (14)	2018	Brazil	Cross-sectional	Polysomnography	40	Mild OSA cohort	49 years	58%	30 kg/m^2^	Mild OSA associated with adverse metabolic profile
Banghøj et al. (16)	2020	Denmark	Randomized controlled trial	Polysomnography, CPAP intervention	72	100% (OSA cohort)	61 years	79%	33 kg/m^2^	CPAP improved glucose variability in some patients
Zamarron et al. (17)	2022	Spain	Randomized clinical trial	Polysomnography	185	100% (OSA cohort)	66 years	72%	34 kg/m^2^	CPAP slowed albuminuria progression

The studies were conducted across multiple geographic regions, including Asia (Saudi Arabia, India, China, Taiwan), Africa (Kenya), Europe (Poland, Denmark, Spain), and South America (Brazil), indicating broad international representation. Sample sizes varied considerably, ranging from relatively small clinical cohorts of fewer than 100 participants to large population-based datasets including several thousand individuals (Table 1). Most studies recruited adult populations with established T2DM, although disease duration and severity varied across studies.

Sleep disorders were assessed using a range of validated instruments and diagnostic approaches. The most commonly used tools included the Pittsburgh Sleep Quality Index (PSQI) for sleep quality assessment and screening questionnaires such as STOP-BANG and the Berlin Questionnaire for obstructive sleep apnea risk. A substantial proportion of studies used objective diagnostic methods, including polysomnography for OSA confirmation, while interventional studies assessed treatment effects of Continuous Positive Airway Pressure (CPAP) therapy. Sleep exposures examined across studies included obstructive sleep apnea, insulin resistance associated with OSA, sleep quality disturbances, and sleep-disordered breathing severity.

The most frequently reported outcomes were glycemic control, particularly HbA1c and glucose variability, followed by prevalence and severity of obstructive sleep apnea. Other outcomes included insulin resistance measures, fasting glucose, and metabolic dysfunction. Diabetes-related complications were also assessed, particularly in relation to renal outcomes. Cardiovascular risk markers were indirectly evaluated in several observational studies, while mental health outcomes and quality of life measures were not a primary focus of the included dataset.

Generally, the included studies demonstrated substantial variability in methodological design, exposure definitions, outcome measurements, and adjustment for confounding factors. Despite this heterogeneity, the studies collectively provided a focused evidence base on the association between obstructive sleep apnea, metabolic dysfunction, and glycemic outcomes in individuals with T2DM, with limited, but emerging evidence from interventional CPAP studies suggesting potential therapeutic benefits.

### 3.1 Risk of bias and quality assessment of included studies

The methodological quality of the included studies was assessed using standardized risk-of-bias tools according to study design (Table 2). Randomized controlled trials were evaluated using the RoB-2 tool, whereas observational studies (cross-sectional and cohort studies) were assessed using the NOS, adapted for cross-sectional studies where appropriate. The NOS evaluates methodological quality across three domains: selection of participants, comparability of study groups, and ascertainment of outcomes/exposures. Overall, the quality of the included studies was variable, reflecting the predominance of observational study designs and heterogeneity in exposure and outcome assessment. Most observational studies were judged to be of moderate to high methodological quality according to NOS criteria, while the randomized controlled trials by Banghøj et al. (16) and Zamarrón et al. (17) demonstrated an overall low risk of bias across most assessed domains. The population-based cohort study by Liu et al. (15) also demonstrated comparatively higher methodological quality than the cross-sectional studies. A key limitation across the evidence base was the widespread reliance on cross-sectional study designs, which inherently limit causal inference and increase susceptibility to selection bias and reverse causality.

**Table 2:** Risk of bias.

Risk domain	Low risk (n)	Some concerns (n)	High risk (n)	Main reason across included studies
Selection bias	3	6	2	Predominance of cross-sectional recruitment and hospital-based or convenience sampling (7–11, 13, 14)
Exposure (sleep disorder measurement)	4	5	2	Mix of polysomnography, validated questionnaires (PSQI, STOP-BANG, Berlin), and self-reported sleep measures
Outcome measurement (HbA1c, metabolic, renal, QoL)	6	4	1	HbA1c and laboratory outcomes objective; QoL and sleep symptoms largely self-reported
Confounding control	3	6	2	Inconsistent adjustment for BMI, diabetes duration, age, comorbid OSA, and medication use
Attrition bias (cohort/RCT only)	2	1	0	Low attrition in RCTs (16, 17); minimal follow-up loss reported
Reporting bias	7	3	1	No protocols for most observational studies; generally consistent outcome reporting

With respect to sleep exposure assessment, substantial variability was observed. Approximately half of the studies used validated screening tools such as the Berlin Questionnaire and STOP-BANG questionnaires (7, 10) or the Pittsburgh Sleep Quality Index (PSQI) (8, 14). Several studies employed objective diagnostic methods, particularly polysomnography for OSA confirmation (11–13), thereby reducing the risk of exposure misclassification. However, questionnaire-based screening without confirmatory testing remained common, particularly in resource-limited settings. The randomized controlled trials (16, 17) used objective clinical assessments and laboratory-based outcome measures, resulting in a lower risk of measurement bias.

Glycemic control outcomes, particularly HbA1c, were generally derived from standardized laboratory measurements, indicating a low risk of outcome measurement bias (12, 16). In contrast, studies relying on self-reported sleep quality or sleep symptoms (8, 14) were more susceptible to reporting bias. Cardiovascular and renal outcomes were variably ascertained, with greater methodological rigor observed in studies using registry-based data or objectively verified clinical endpoints (15, 17) than in those relying primarily on clinical history or self-reported outcomes.

Control for confounding varied across the included studies. Although most adjusted for major confounders such as age, sex, and body mass index, fewer studies comprehensively accounted for diabetes duration, medication use, socioeconomic status, lifestyle factors, or coexisting sleep disorders. Consequently, residual confounding remained a concern, particularly among the cross-sectional studies. The cohort study and randomized controlled trials generally demonstrated more robust adjustment for potential confounders; however, unmeasured confounding could not be completely excluded. This was reflected in the NOS comparability domain, where several studies received lower scores because of limited adjustment for important confounding variables (Supplementary Table S2).

Selective reporting bias was difficult to evaluate because study protocols were unavailable for most observational studies. Nevertheless, there was no clear evidence of systematic selective outcome reporting, as the reported outcomes were generally consistent with the stated study objectives. Overall, the body of evidence was considered to be of moderate methodological quality, with confidence in the findings strengthened by the inclusion of higher-quality cohort and randomized controlled studies (15–17) and by the use of objective sleep assessment methods in several investigations. However, the predominance of cross-sectional studies, variability in exposure and outcome measurement, and inconsistent adjustment for confounding factors should be considered when interpreting the overall findings.

### 3.2 Prevalence of sleep disorders in T2DM

Sleep disorders were highly prevalent among individuals with T2DM, although estimates varied considerably depending on the population studied, diagnostic approach, and definition of sleep disturbance. Overall, OSA emerged as the most frequently investigated condition, followed by sleep quality disturbances and abnormal sleep-related metabolic profiles.

Reported prevalence of OSA in individuals with T2DM varied widely across studies, largely reflecting differences in diagnostic methods. Studies using polysomnography generally reported higher and more precise prevalence estimates compared with those relying on screening questionnaires such as the STOP-BANG or Berlin Questionnaire (7, 10). Across studies, a substantial proportion of patients with T2DM were identified as being at intermediate to high risk of OSA, with several cohorts reporting that up to approximately 40–60% in some cohorts met criteria suggestive of clinically significant sleep-disordered breathing (9, 11).

In addition to diabetic populations, studies in nondiabetic or mixed metabolic cohorts demonstrated that OSA was also highly prevalent in individuals with metabolic dysfunction and insulin resistance. For instance, both Li et al. (12), and Michalek-Zrabkowska et al. (13), confirmed that OSA severity was common in overweight and obese populations and was associated with adverse metabolic phenotypes, suggesting that sleep-disordered breathing is strongly linked to metabolic dysregulation even before the onset of diabetes.

Poor sleep quality, most commonly assessed using the PSQI, was reported in studies such as Sokwalla et al. (8) and Silva et al. (14), where a large proportion of participants demonstrated poor sleep quality. Although exact pooled prevalence estimates were not consistently reported across all studies, findings indicated that poor sleep quality was common among individuals with T2DM and was frequently associated with worse metabolic outcomes. Similarly, sleep duration abnormalities were also observed across several studies, although this was not a primary exposure in most included research. Short sleep duration was more consistently associated with adverse metabolic profiles, including insulin resistance and worse glycemic control, as demonstrated in observational findings embedded within larger metabolic studies (12, 14). Excessive daytime sleepiness was not systematically assessed across all studies, but when reported, it was identified as a clinically relevant symptom in individuals with OSA and T2DM, particularly in studies using symptom-based screening tools.

Interestingly, the findings from the included studies consistently demonstrate that sleep disorders are highly prevalent in individuals with T2DM, with OSA representing the most frequently investigated and clinically significant condition. However, variability in diagnostic approaches (polysomnography versus questionnaire-based screening), study populations, and reporting methods contributed to heterogeneity in prevalence estimates across studies.

### 3.3 Association between sleep disorders and glycemic control

Across the included studies, a consistent association was observed between sleep disorders and poorer glycemic control in individuals with T2DM, although the magnitude of this relationship varied depending on the type and severity of sleep disturbance, as well as study design and adjustment for confounders. OSA was the most frequently studied sleep disorder in relation to glycemic outcomes. The majority of studies reported higher HbA1c levels, impaired glucose regulation, or worse metabolic profiles among individuals with OSA compared with those without sleep-disordered breathing (7, 9, 11). Several studies also demonstrated a severity-dependent relationship, where increasing OSA severity, typically assessed by the apnea-hypopnea index (AHI), was associated with progressively worse glycemic control and insulin resistance (11, 12). These associations generally remained significant after adjustment for key covariates such as age, sex, and body mass index, although attenuation of effect estimates was observed in some studies following multivariable adjustment.

In addition, mechanistic and non-diabetic cohort studies further supported these findings. Li et al., demonstrated that OSA was associated with glucose dysmetabolism and pancreatic *β*-cell dysfunction in overweight, and obese young adults without diabetes, suggesting a potential causal pathway linking sleep-disordered breathing and impaired glucose regulation (12). Similarly, Michalek-Zrabkowska et al., reported that OSA was independently associated with insulin resistance in nondiabetic adults, reinforcing the metabolic impact of sleep fragmentation and intermittent hypoxia (13).

However, sleep quality disturbances were indirectly associated with glycemic outcomes in studies using validated screening tools. For instance, Sokwalla et al., reported that poorer sleep quality in individuals with T2DM was associated with worse overall metabolic control and higher cardiometabolic risk profiles (8). Silva et al., further supported these findings by demonstrating that even mild sleep disturbance was associated with adverse metabolic parameters, although HbA1c was not consistently reported across all studies assessing sleep quality (14).

Sleep-disordered breathing severity rather than subjective sleep complaints appeared to be more strongly associated with glycemic dysregulation. This was particularly evident in studies using objective polysomnography, where stronger associations between OSA severity and metabolic dysfunction were observed (11, 12). Despite variability in measurement approaches and adjustment for confounding factors, the evidence consistently indicates that sleep-disordered breathing, particularly OSA is associated with poorer glycemic control and adverse metabolic outcomes in individuals with T2DM. However, the strength of evidence for sleep quality and sleep duration is less consistent, due to limited and heterogeneous reporting across studies.

### 3.4 Sleep disorders and diabetes-related complications

The included studies showed that sleep disorders were consistently associated with an increased burden of diabetes-related complications in individuals with T2DM, although the strength of these associations varied depending on study design, outcome definition, and sleep disorder measurement, with OSA as the most frequently examined sleep disorder in relation to diabetes-related complications. Several studies reported that increasing OSA severity was associated with worse cardiometabolic profiles and higher burden of diabetes-related comorbidities. In particular, Zhang et al., demonstrated that hospitalized patients with T2DM and OSA exhibited more severe metabolic dysfunction, including insulin resistance and adverse cardiovascular risk profiles (11). Similarly, the population-based cohort by Liu and Wu., supported a bidirectional relationship between sleep apnea and diabetes, suggesting that sleep-disordered breathing may contribute to the development and progression of diabetes-related complications over time (15).

Microvascular complications were also indirectly evaluated in several studies through metabolic and renal outcomes. Zamarrón et al., demonstrated that patients with T2DM and OSA who received CPAP therapy had slower progression of albuminuria, suggesting a potential protective effect of treating sleep-disordered breathing on diabetic kidney disease (17). This provides evidence that OSA may contribute to the progression of renal complications in diabetes and that intervention may modify this risk. In addition, insulin resistance and metabolic dysfunction, key drivers of diabetes-related complications, were consistently associated with OSA severity in mechanistic and observational studies. Li et al., showed that OSA was associated with impaired *β*-cell function and worsening glucose metabolism (15), while Michalek-Zrabkowska et al., demonstrated that OSA independently contributed to insulin resistance in nondiabetic adults (13). These findings support a pathophysiological link between sleep-disordered breathing and downstream diabetic complications.

Sleep quality disturbances were less frequently assessed in relation to specific diabetes complications, but were indirectly associated with worse metabolic profiles in studies such as Sokwalla et al., and Silva et al. These studies suggested that poor sleep quality may contribute to an increased cardiometabolic risk burden in individuals with T2DM, although direct complication endpoints were not consistently reported (8, 14). Abnormalities in sleep duration were not systematically examined as direct predictors of diabetes complications across the included studies. However, mechanistic evidence from metabolic studies by Li et al., and Silva et al., suggests that short sleep duration may contribute to insulin resistance and metabolic dysregulation, which are key mediators of microvascular and macrovascular diabetic complications (12, 14).

Although heterogeneity in outcome definitions limited direct comparability across studies, the evidence from the included literature suggests that sleep disorders, particularly OSA, are associated with a higher burden of diabetes-related complications. The strongest evidence supports an association between OSA and progression of metabolic and renal dysfunction, with emerging evidence suggesting that treatment of OSA (e.g., CPAP therapy) may slow complication progression in T2DM populations.

### 3.5 Sleep disorders and cardiovascular outcomes

Across the included studies, sleep disorders were consistently associated with an increased cardiovascular risk profile in individuals with T2DM, although the magnitude and consistency of these associations varied by sleep disorder type, outcome definition, and study design. Several observational studies demonstrated that OSA was associated with adverse cardiometabolic profiles in individuals with T2DM. In particular, Zhang et al., reported that hospitalized patients with T2DM and OSA exhibited more severe metabolic dysfunction and higher prevalence of cardiometabolic risk factors, including obesity and insulin resistance, which are key contributors to cardiovascular disease risk (11). Similarly, Kalakattawi et al., and Viswanathan et al., reported that a substantial proportion of individuals with T2DM were at high risk of OSA, a condition strongly linked to hypertension and cardiovascular morbidity in diabetic populations (7, 9).

Mechanistic and population-based evidence further supported these findings, with Liu and Wu et al., demonstrated a bidirectional relationship between sleep apnea and diabetes, suggesting shared pathophysiological pathways contributing to both metabolic and cardiovascular disease progression (15). Michalek-Zrabkowska et al., also reported that OSA was independently associated with insulin resistance, a key intermediate pathway linking sleep-disordered breathing and cardiovascular risk (13). Also, CPAP intervention studies provided additional indirect evidence of cardiovascular benefit. For instance, Zamarrón et al., demonstrated that treatment of OSA with CPAP in patients with T2DM and diabetic kidney disease slowed the progression of albuminuria, a marker of systemic vascular damage and cardiovascular risk (17). Although direct cardiovascular endpoints were not assessed in all studies, this finding suggests a potential protective effect of treating sleep-disordered breathing on vascular outcomes.

While, the duration of sleep abnormalities and subjective sleep disturbances were not consistently evaluated in relation to hard cardiovascular endpoints, metabolic studies such as Li et al., and Silva et al., indicated that short sleep duration and sleep fragmentation were associated with insulin resistance and adverse metabolic profiles, which are established risk factors for cardiovascular disease (12, 14). Similarly, Sokwalla et al., reported that poor sleep quality in individuals with T2DM was associated with worse overall health status and increased cardiometabolic risk burden, although direct cardiovascular outcomes were not systematically measured (8). Despite, high heterogeneity in outcome definitions and limited direct measurement of cardiovascular events restricted comparability, the evidence from the included studies consistently suggests that sleep disorders, particularly OSA are associated with an adverse cardiometabolic risk profile in individuals with T2DM, supporting a clinically important link between sleep-disordered breathing and cardiovascular risk pathways in diabetic populations.

### 3.6 Sleep disorders and quality of life outcomes

Some of the included studies reported significant associations between sleep disorders and impaired quality of life, reduced functional status, and adverse psychological well-being among individuals with T2DM, although the number of studies directly assessing these outcomes was limited within the included evidence base. For example, Sokwalla et al., reported that individuals with T2DM and poor sleep quality experienced greater sleep-related impairment and reduced overall well-being compared with those without significant sleep disturbance (8). Similarly, Silva et al., demonstrated that individuals with mild obstructive sleep apnea exhibited worse metabolic and functional profiles, suggesting that even early-stage sleep disturbance may contribute to reduced health status in diabetic populations (14).

Excessive daytime sleepiness and functional impairment were not systematically assessed across all included studies; however, findings from OSA-related cohorts suggested that sleep-disordered breathing is associated with reduced daytime functioning. In particular, Zhang et al., reported that patients with T2DM and OSA exhibited more severe metabolic dysfunction and poorer overall health status, which may indirectly contribute to reduced quality of life and impaired daily functioning (11).

Furthermore, OSA was indirectly associated with psychological burden and reduced health status, with Kalakattawi et al., and Viswanathan et al., identified a high prevalence of OSA risk in individuals with T2DM, a condition known to be associated with fatigue, reduced energy levels, and impaired functional capacity (7, 9). In addition, Liu and Wu et al., suggested a bidirectional relationship between sleep apnea and diabetes, indicating that chronic sleep-disordered breathing may contribute to a cycle of worsening metabolic and psychological health outcomes (15). Although CPAP intervention studies primarily focused on metabolic and renal outcomes, Zamarrón et al., suggested that treatment of OSA may improve overall health status by reducing disease burden associated with diabetic kidney disease, which may indirectly enhance quality of life in affected individuals (17).

### 3.7 Subgroup analyses

Subgroup analyses were conducted to explore potential sources of heterogeneity across the included studies and to assess whether associations between sleep disorders and outcomes in T2DM differed according to sleep disorder type, study design, geographic region, and assessment methodology (Table 3).

**Table 3:** Subgroup analysis.

Subgroup category	Characteristics of included studies	Main findings	Overall interpretation
Type of sleep disorder: Obstructive sleep apnea (OSA)	Majority of studies; assessed using polysomnography (7, 9–13)	Consistently associated with poorer glycemic control, insulin resistance, and adverse cardiometabolic profiles	Strongest and most consistent evidence across all sleep disorder categories
Type of sleep disorder: Poor sleep quality	Assessed using PSQI in Sokwalla et al. (8) and Silva et al. (14)	Associated with poorer metabolic profiles, fatigue, and reduced health status	Moderate but consistent association with adverse metabolic and functional outcomes
Type of sleep disorder: Insomnia symptoms	Not directly assessed as a primary exposure in included studies	Indirect associations with fatigue, sleep fragmentation, and impaired function reported in broader sleep quality studies	Evidence limited and indirect due to lack of dedicated insomnia studies
Type of sleep disorder: Sleep duration abnormalities	Examined in metabolic/OSA-related studies (12, 14)	Short sleep duration associated with worse metabolic profiles and insulin resistance; long sleep inconsistently reported	Short sleep shows more consistent associations than long sleep duration
Study design: Cross-sectional studies	Majority of included studies (7–11, 13, 14)	Consistent associations between sleep disorders and adverse glycemic and metabolic outcomes	Strong associative evidence but limited causal inference
Study design: Cohort/analytical studies	Liu and Wu (15) bidirectional cohort; Li et al. (12) mechanistic observational study	Demonstrated bidirectional and mechanistic links between OSA and metabolic dysfunction	Provided temporal and mechanistic support for associations
Study design: Randomized controlled trials	(16, 17)	CPAP improved glycemic monitoring outcomes and slowed renal disease progression	Provides interventional evidence supporting clinical relevance of OSA treatment
Geographic region: Asia/Middle East	(7, 9, 11)	High prevalence of OSA risk; consistent associations with metabolic outcomes	Findings consistent despite reliance on screening tools
Geographic region: Europe	(10, 13, 16, 17)	More frequent use of objective sleep and metabolic assessments	Methodologically stronger evidence base
Sleep assessment method: Objective measures	Polysomnography and CGM-based studies (11–13, 16)	Stronger and more consistent associations with metabolic outcomes	Lower risk of exposure misclassification
Sleep assessment method: Self-reported/questionnaire-based	(7–10, 14)	Greater variability in prevalence and effect estimates	More susceptible to measurement and reporting bias
Adjustment for confounding variables	Variable across studies; most adjusted for age, sex, BMI; fewer adjusted for diabetes duration or OSA severity	Adjusted analyses showed attenuated but persistent associations	Residual confounding likely influences magnitude of associations

#### 3.7.1 By type of sleep disorder

The strongest and most consistent associations with adverse metabolic outcomes were observed in studies investigating OSA. Across the included studies, OSA particularly when assessed using polysomnography or validated screening tools was consistently associated with poorer glycemic control, insulin resistance, and adverse cardiometabolic profiles (11–13). In contrast, studies examining sleep quality disturbances (e.g., PSQI-based assessments) showed more moderate, but still significant associations with metabolic outcomes and overall health status (8, 14). Sleep duration was not a primary exposure in most included studies; however, where assessed, short sleep duration was more consistently associated with adverse metabolic profiles compared with long sleep duration (12, 14).

#### 3.7.2 By study design

Both of the randomized controlled trials by Banghøj et al., and Zamarrón et al., as well as the large population-based cohort study Liu & Wu et al., generally provided more robust and temporally informative evidence compared with cross-sectional studies (15–17). Cross-sectional studies consistently demonstrated associations between sleep disorders and adverse metabolic outcomes, but were more susceptible to reverse causality and residual confounding (7–11, 13, 14). Overall, stronger and more clinically meaningful associations were observed in studies with longitudinal or interventional designs, particularly for metabolic and renal outcomes (15).

#### 3.7.3 By geographic region

Regional variation in study design and diagnostic methodology was evident, such as that studies conducted in Asia and the Middle East more commonly relied on screening questionnaires or hospital-based cohorts, whereas European studies frequently used objective diagnostic approaches such as polysomnography or continuous glucose monitoring. Despite these methodological differences, the direction of associations between sleep disorders and adverse metabolic outcomes remained broadly consistent across regions.

#### 3.7.4 By sleep assessment method

Studies using objective diagnostic methods, particularly polysomnography, consistently reported stronger and more robust associations between OSA severity and metabolic dysfunction than studies relying on questionnaire-based screening tools. For example, Zhang et al. and Michalek-Zrabkowska et al. demonstrated clear relationships between objectively measured OSA severity and poorer glycemic control. In contrast, questionnaire-based studies, including those by Kalakattawi et al. and Westlake et al., reported more variable findings, with differences in effect estimates likely reflecting the lower sensitivity and specificity of screening questionnaires for identifying OSA. Furthermore, interventional studies incorporating objective physiological monitoring, such as Banghøj et al., provided additional evidence that treatment of sleep-disordered breathing can improve metabolic outcomes. Overall, the findings suggest that studies using objective sleep assessment methods produce more consistent evidence linking OSA with impaired glycemic control, whereas questionnaire-based assessments are more prone to measurement error and misclassification, contributing to greater variability across studies.

#### 3.7.5 By adjustment for confounding variables

Studies that adjusted for major confounders such as age, sex, and body mass index generally reported attenuated but still significant associations between sleep disorders and metabolic outcomes. This was particularly evident in cross-sectional studies examining OSA risk and glycemic outcomes (7–9). In contrast, studies with limited adjustment often reported stronger effect estimates, suggesting potential residual confounding. Hence, adjustment for key metabolic and demographic variables varied considerably across studies.

In general, subgroup analyses demonstrated that the associations between sleep disorders and adverse outcomes in T2DM were broadly consistent across study designs and populations. However, effect sizes varied according to sleep disorder type, diagnostic method, and extent of confounder adjustment, with the most robust and consistent findings observed for obstructive sleep apnea assessed using objective diagnostic methods.

## 4 Discussion

This systematic review examined the relationship between OSA, sleep disturbances, and glucose dysregulation in adults with T2DM. Overall, the findings demonstrate a consistent association between sleep-disordered breathing, particularly OSA and adverse metabolic outcomes, including poorer glycemic control, increased insulin resistance, and a higher burden of diabetes-related complications. Although heterogeneity in study design, exposure assessment, and outcome measurement was substantial, the directionality of associations remained broadly consistent across populations and settings. The observed associations are consistent with the broader literature linking sleep disruption and metabolic dysfunction. Experimental sleep restriction studies in healthy adults have demonstrated reduced insulin sensitivity after only a few nights of curtailed sleep (18). Similarly, chronic sleep fragmentation has been shown to activate inflammatory pathways implicated in metabolic syndrome development (19). These mechanistic studies reinforce the biological plausibility of the associations observed in the present review.

A key finding of this review is the strong and reproducible association between OSA and impaired metabolic control in T2DM. Most included studies reported higher HbA1c levels, worse insulin sensitivity, or more adverse metabolic profiles among individuals with OSA compared with those without sleep-disordered breathing. These observations align with large epidemiological evidence demonstrating that OSA is an independent risk factor for incident T2DM. For example, the Sleep Heart Health Study reported that severe OSA was associated with approximately a twofold increased risk of developing diabetes, independent of adiposity and other confounders (20). Similarly, a meta-analysis of longitudinal studies by Reutrakul et al., confirmed a significant association between OSA and incident T2DM, supporting the directionality suggested by the present study (21). Mechanistically, intermittent hypoxia and sleep fragmentation in OSA are thought to drive metabolic dysfunction through sympathetic activation, systemic inflammation, and oxidative stress, all of which impair insulin signaling pathways.

At the molecular level, intermittent hypoxia induces repeated cycles of hypoxia-reoxygenation that resemble ischemia–reperfusion injury, resulting in excessive generation of reactive oxygen species (ROS) from mitochondrial electron transport chains and activation of nicotinamide adenine dinucleotide phosphate (NADPH) oxidases (22). The resulting oxidative stress activates several redox-sensitive signaling pathways, including nuclear factor-κB (NF-κB), c-Jun N-terminal kinase (JNK), and p38 mitogen-activated protein kinase (MAPK), leading to increased production of pro-inflammatory cytokines such as tumor necrosis factor-*α* (TNF-α), interleukin-6 (IL-6), and C-reactive protein (CRP) (23, 24). These inflammatory mediators promote serine phosphorylation of insulin receptor substrate-1 (IRS-1), thereby impairing insulin receptor signaling, reducing phosphatidylinositol 3-kinase (PI3K)/Akt activation, and limiting glucose transporter type 4 (GLUT4) translocation in skeletal muscle and adipose tissue (22). Consequently, peripheral glucose uptake is diminished, contributing to insulin resistance and poor glycemic control. Furthermore, oxidative stress impairs pancreatic *β*-cell function, which is particularly vulnerable to oxidative damage because of its relatively low endogenous antioxidant capacity, thereby reducing insulin secretion and further exacerbating glucose dysregulation. Intermittent hypoxia also stabilizes hypoxia-inducible factor-1α (HIF-1α), promoting metabolic reprogramming, adipose tissue inflammation, endothelial dysfunction, and sympathetic nervous system activation, all of which contribute to the increased cardiovascular and metabolic risk observed in patients with OSA and T2DM (22–24). Experimental models of intermittent hypoxia have consistently demonstrated reduced insulin sensitivity and impaired glucose tolerance, even in the absence of obesity, providing biological support for these mechanisms (22). These mechanisms likely explain the consistent dose–response relationship observed in several included studies, whereby increasing OSA severity correlated with worsening glycemic control.

Our findings are consistent with previous studies that have reported similar associations between sleep-disordered breathing and metabolic dysregulation. For example, a meta-analysis by Iftikhar et al., found that CPAP therapy modestly improves insulin sensitivity, particularly in patients with severe OSA, although effects on HbA1c were less consistent (23). More recently, Song et al., highlighted that metabolic improvements following CPAP are heterogeneous and may depend on adherence, baseline glycemic status, and obesity phenotype (24). This may explain why the included randomized controlled trials in this review demonstrated beneficial effects on intermediate metabolic or renal outcomes, but did not uniformly show large improvements in glycemic control (16, 17).

Importantly, the findings from this study extend beyond OSA to other sleep disturbances such as poor sleep quality and short sleep duration, which were also associated with adverse metabolic outcomes, albeit less consistently. Large population-based studies have previously demonstrated a U-shaped relationship between sleep duration and diabetes risk, with both short and long sleep associated with increased incidence of T2DM (25). The present review supports these findings, but highlights that sleep duration remains under-investigated in diabetic populations, limiting firm conclusions.

Nevertheless, an important contribution of this review is the synthesis of evidence linking OSA with diabetes-related complications, particularly renal and cardiometabolic outcomes. For example, Zamarrón et al., demonstrated that CPAP therapy slowed progression of albuminuria in patients with T2DM and OSA, suggesting a potential disease-modifying effect (17). This aligns with observational evidence indicating that OSA is independently associated with chronic kidney disease progression and microvascular dysfunction (5).

However, evidence for macrovascular outcomes remains less consistent, with some cohort studies suggest increased cardiovascular risk in OSA populations (26, 27), others have reported attenuation of associations after full adjustment for obesity and metabolic syndrome components (28, 29). This highlights an ongoing challenge in disentangling the independent effect of OSA from shared cardiometabolic risk factors.

While OSA may contribute to glucose dysregulation, T2DM may also exacerbate OSA severity through neuropathic and microvascular mechanisms affecting upper airway control (30). Diabetic neuropathy has been shown to impair pharyngeal muscle responsiveness, potentially worsening airway collapsibility during sleep (31). In addition, hyperglycemia-related oxidative stress may further contribute to ventilatory instability. This bidirectional relationship complicates causal inference and highlights the importance of longitudinal and interventional studies.

Clinically, the finding from this review suggests the screening for OSA in patients with T2DM may be justified, particularly in individuals with poor glycemic control or resistant hypertension. While, current clinical guidelines already recommend consideration of sleep evaluation in high-risk diabetic populations, implementation remains inconsistent in practice. Equally, the modest metabolic benefits observed with CPAP therapy, suggest that while OSA treatment may improve intermediate physiological outcomes, it should not be considered a standalone intervention for glycemic control. Instead, a multidisciplinary approach targeting weight reduction, lifestyle modification, and metabolic optimization is likely required.

### 4.1 Strengths and limitations of the evidence

A major strength of this review is the inclusion of diverse study designs across multiple geographic regions, including cross-sectional studies, cohort data, and randomized controlled trials. This allows a broad evaluation of associations across different levels of evidence. However, several limitations should be acknowledged. First, the predominance of observational and cross-sectional studies among the included evidence may limit causal inference. Second, exposure definitions varied widely, ranging from polysomnography to self-reported questionnaires, introducing substantial measurement heterogeneity. Third, confounding adjustment was inconsistent, particularly for key variables such as obesity, diabetes duration, and medication use (32). These factors likely contributed to variability in effect estimates. Additionally, differences in study populations, sample sizes, diagnostic criteria for sleep disorders, and outcome measures for glycemic control further limited comparability across studies. From the perspective of the review itself, the relatively small number of eligible studies and the substantial clinical and methodological heterogeneity precluded quantitative meta-analysis. Furthermore, restricting inclusion to studies published in English may have introduced language bias, and publication bias cannot be excluded despite the comprehensive search strategy.

### 4.2 Future directions

Future research should prioritize longitudinal cohort studies with repeated objective sleep assessments and detailed metabolic phenotyping. Randomized controlled trials with adequate CPAP adherence monitoring and longer follow-up are needed to clarify whether treating OSA can meaningfully improve hard metabolic and cardiovascular endpoints in T2DM. In addition, emerging technologies such as continuous glucose monitoring may provide more sensitive assessments of glycemic variability in relation to sleep disturbances.

## 5 Conclusion

In summary, this systematic review provides evidence of a consistent association between OSA and adverse metabolic outcomes in individuals with T2DM, with additional, but less robust associations observed for other sleep disturbances. While causality cannot be definitively established, the convergence of epidemiological, mechanistic, and interventional evidence supports a clinically meaningful link between sleep-disordered breathing and glucose dysregulation. These findings highlight the importance of integrated management strategies addressing both sleep and metabolic health in patients with T2DM.
