# Supplementary material for: Obstructive sleep apnoea and glucose dysregulation in type 2 diabetes: a systematic review of the literature
Source: Front Med (Lausanne). 2026 Sep 7;13:1915032. doi: 10.3389/fmed.2026.1915032 (PMC13592258; doi:10.3389/fmed.2026.1915032)
Supplement: Supplementary file 1 [file Table_1.DOCX]

**Supplementary Table 1. Search strategy**

| **Concept** | **Keywords/MeSH Terms** | **Example Search String** |
| --- | --- | --- |
| Sleep disorders | “sleep disorder*”, “sleep disturbance*”, “sleep quality”, “sleep deprivation”, insomnia, “obstructive sleep apnea”, “sleep apnea”, “restless legs syndrome”, circadian, “sleep wake disorders” | (“Sleep Wake Disorders”[Mesh] OR “sleep disorder*” OR “sleep disturbance*” OR insomnia OR “obstructive sleep apnea” OR “sleep apnea” OR “restless legs syndrome” OR circadian OR “sleep quality” OR “sleep deprivation”) |
| Type 2 diabetes | “type 2 diabetes mellitus”, T2DM, “non-insulin dependent diabetes”, “diabetes mellitus type 2” | (“Diabetes Mellitus, Type 2”[Mesh] OR “type 2 diabetes mellitus” OR T2DM OR “non-insulin dependent diabetes” OR “diabetes mellitus type 2”) |
| Outcomes/associations | prevalence, incidence, association, correlation, “glycemic control”, HbA1c, complications, “quality of life”, depression, mortality | (prevalence OR incidence OR association OR correlation OR “glycemic control” OR HbA1c OR complications OR “quality of life” OR depression OR mortality) |
| Study type | “systematic review”, “meta-analysis”, observational, cohort, cross-sectional, case-control”, randomized controlled trials” | (“systematic review” OR “meta-analysis” OR observational OR cohort OR “cross-sectional” OR “case-control” OR “randomized controlled trials”) |
| Combined search strategy | Combination of all concepts using Boolean operators | ((“Sleep Wake Disorders”[Mesh] OR “sleep disorder*” OR insomnia OR “obstructive sleep apnea” OR “sleep apnea” OR “restless legs syndrome” OR “sleep quality” OR circadian) AND (“Diabetes Mellitus, Type 2”[Mesh] OR “type 2 diabetes mellitus” OR T2DM OR “non-insulin dependent diabetes”) AND (prevalence OR association OR “glycemic control” OR HbA1c OR complications OR “quality of life”)) |

**Supplementary Table S2. Quality assessment of included studies**

| **Study** | **Design** | **Tool** | **Score / Judgment** | **Overall Quality** |
| --- | --- | --- | --- | --- |
| Kalakattawi et al. | Cross-sectional | NOS | 6/10 | Moderate |
| Sokwalla et al. | Cross-sectional | NOS | 7/10 | High |
| Viswanathan et al. | Cross-sectional | NOS | 6/10 | Moderate |
| Zhang et al. | Cross-sectional | NOS | 8/10 | High |
| Liu & Wu | Cohort | NOS | 8/10 | High |
| Li et al. | Cross-sectional | NOS | 7/10 | High |
| Michalek-Zrabkowska et al. | Cross-sectional | NOS | 7/10 | High |
| Silva et al. | Cross-sectional | NOS | 6/10 | Moderate |
| Banghøj et al. | RCT | RoB-2 | Low risk | High |
| Zamarrón et al. | RCT | RoB-2 | Some concerns | Moderate-High |
